# Identification of a four-gene panel predicting overall survival for lung adenocarcinoma

**DOI:** 10.1186/s12885-020-07657-9

**Published:** 2020-12-07

**Authors:** Chunyu Li, Qizhong Long, Danni Zhang, Jun Li, Xianming Zhang

**Affiliations:** Department of Respiratory and Critical Care Medicine, Affiliated Hospital of Guizhou Medical University, Guiyang, 550004 China

**Keywords:** Lung adenocarcinoma, Biomarkers, Four-gene panel, Prognosis, GNG7, DNA methylation

## Abstract

**Background:**

Lung cancer is the most frequently diagnosed carcinoma and the leading cause of cancer-related mortality. Although molecular targeted therapy and immunotherapy have made great progress, the overall survival (OS) is still poor due to a lack of accurate and available prognostic biomarkers. Therefore, in this study we aimed to establish a multiple-gene panel predicting OS for lung adenocarcinoma.

**Methods:**

We obtained the mRNA expression and clinical data of lung adenocarcinoma (LUAD) from TCGA database for further integrated bioinformatic analysis. Lasso regression and Cox regression were performed to establish a prognosis model based on a multi-gene panel. A nomogram based on this model was constructed. The receiver operating characteristic (ROC) curve and the Kaplan–Meier curve were used to assess the predicted capacity of the model. The prognosis value of the multi-gene panel was further validated in TCGA-LUAD patients with EGFR, KRAS and TP53 mutation and a dataset from GEO. Gene set enrichment analysis (GSEA) was performed to explore potential biological mechanisms of a novel prognostic gene signature.

**Results:**

A four-gene panel (including DKK1, GNG7, LDHA, MELTF) was established for LUAD prognostic indicator. The ROC curve revealed good predicted performance in both test cohort (AUC = 0.740) and validation cohort (AUC = 0.752). Each patient was calculated a risk score according to the model based on the four-gene panel. The results showed that the risk score was an independent prognostic factor, and the high-risk group had a worse OS compared with the low-risk group. The nomogram based on this model showed good prediction performance. The four-gene panel was still good predictors for OS in LUAD patients with TP53 and KRAS mutations. GSEA revealed that the four genes may be significantly related to the metabolism of genetic material, especially the regulation of cell cycle pathway.

**Conclusion:**

Our study proposed a novel four-gene panel to predict the OS of LUAD, which may contribute to predicting prognosis accurately and making the clinical decisions of individual therapy for LUAD patients.

## Background

Lung cancer is the most frequently diagnosed carcinoma and the leading cause of cancer-related mortality worldwide, with 2.1 million new lung cancer cases and 1.8 million deaths predicted in 2018 [1]. More than 80% of lung cancers are non-small cell lung cancer, mainly lung adenocarcinoma and lung squamous cell carcinoma [2]. Among them, lung adenocarcinoma is on the rise and occupies the main part gradually [3–5]. Traditional treatments for NSCLC included surgery, chemotherapy, and radiotherapy. Although molecular targeted therapy and immunotherapy for NSCLC (especially lung adenocarcinoma) have made great progress in recent years, the OS of NSCLC is still poor, with a 5-year OS of less than 18% [6]. Hence, the identification of accurate prognostic biomarkers and novel and effective therapeutic targets remains particularly urgent for improving the poor survival of NSCLC patients.

Recent advances in genome-wide technologies have promoted the development of tumor biomarkers studies. Large numbers of biomarkers related to diagnosis, prognosis, and drug resistance of cancers have been detected. However, many studies were confined to a single biomarker or a small sample cohort, which made the accuracy and availability of biomarkers insufficient. Therefore, the combination of multiple biomarkers and large sample analysis is more promising. For example, Liu et al. established a six-gene signature prognostic model (including CSE1L, CSTB, MTHFR, DAGLA, MMP10, and GYS2) using data from The Cancer Genome Atlas-Liver Hepatocellular Carcinoma Dataset (TCGA-LIHC) [7]. Mining of novel and reliable gene prognostic markers contribute to the prognosis risk stratification and precision therapy of cancer patients.

In the present study, we performed lasso regression, univariate Cox regression, and multivariate Cox regression analysis to screen novel prognostic biomarkers and established a multi-gene panel as a prognostic indicator using data from TCGA-LUAD. ROC curve and Kaplan–Meier curve were used to estimate the prognostic performance of the multi-gene panel. Then, prognosis value of the multi-gene panel was further validated using a dataset from GEO database. Furthermore, we further investigated the clinical significance and possible biological functions of one of the key gene signatures. Overall, our results indicated that the four-gene panel might contribute to predicting OS of LUAD patients effectively and might become a novel target for precision therapy.

## Methods

### Identification of differentially expressed mRNA in LUAD

The mRNA expression and clinical data were downloaded from the TCGA Database (LUAD mRNA expression (IlluminaHiseq), containing 497 LUAD samples and 54 normal samples). Raw expression data underwent a log2 transformation. Differential expression genes (DEGs) were screened via using limma package in R version 3.5.3 [8]. DEGs were defined according to the criterion: |logFC| > 1, FDR < 0.05.

### Establishment of the prognostic gene panel

The genes associated with the OS for LUAD patients were identified using Univariate Cox regression analysis, with a cut-off of *P* < 0.001 being considered significant. Lasso penalized regression analysis was utilized to further narrow the range of prognostic genes [9]. Then a prognostic risk model of gene panel was set up based on a linear combination of the multivariate Cox regression model coefficients (β) multiplied with its mRNA expression value. The risk score = (βmRNA1 * expression value of mRNA1) + (βmRNA2 * expression value of mRNA2) + (βmRNA3 * expression value of mRNA3) + ⋯ + (βmRNAn * expression value of mRNAn). Each patient was calculated a risk score according to this model. Then we divided these patients into a high-risk group and a low-risk group according to a cut-off value calculated via the R package “survminer” and “survival” and two-sided log-rank test. The predictive performance of the gene panel for OS was estimated using a time-dependent ROC curve by the “survivalROC” package in R software [10]. The Kaplan–Meier survival curve was executed to compare the survival difference in the high- and low-risk cohort by the “survival” package in R software.

### Validation of the prognostic gene panel

To further validate the prognostic value of the gene panel, GSE42127 data from the GEO database were downloaded [11]. The gene expression of GSE42127 data and TCGA-LUAD data were uniformly corrected using the R package “sva” to make them comparable. The risk score was computed with the gene-panel model for each included patient. The Kaplan–Meier curve and ROC curve were performed to validate the predictive capacity of the prognostic gene panel.

### The four-gene panel is an independent prognostic factor for LUAD

Univariate and multivariate Cox regression analyses with forwarding stepwise procedure were performed to investigate whether the four-gene panel could be an independent prognostic factor for LUAD patients. Clinical parameters included including gender, age, TNM stage.

### Establishment of a predictive nomogram

Nomogram, a simple data evaluation model for the probability of an event, is often used to predict tumor prognosis [12]. Clinical parameters and risk scores from TCGA-LUAD patients were used to build a nomogram in the R package “rms” to detect the predictive probability of 1-year, 3-year, and 5-year OS for LUAD. The discrimination of the nomogram was assessed by using the concordance index (C-index) with a bootstrap method. The calibration curve of the nomogram was plotted by calibrating function of R software to compare predicted OS against observed OS.

### MethHC database

MethHC (A database of DNA Methylation and gene expression in Human Cancer) is an online analysis web based on TCGA database resource focused on the DNA methylation of human diseases. We explored DNA methylation level and mRNA expression of GNG7 using MethHC (http://methhc.mbc.nctu.edu.tw/) [13].

### GSEA

To explore potential biological mechanisms of prognostic gene signature expression on LUAD prognosis, GSEA was used to investigate the enrichment of a priori defined set of genes between the high- and low-expression groups [14]. Gene sets enriched significantly were screened according to the criterion: a normal *P*-value < 0.05.

### Statistical analysis

Univariate Cox regression, lasso regression, multivariate Cox regression analysis, Kaplan–Meier curve, the ROC curve, and log-rank test were used in the present study. All statistical analyses and the generation of relevant figures were operated by R software version 3.5.3. The statistical significance was established at *P* < 0.05.

## Results

### Identification of DEGs in LUAD

**Fig. 1:**
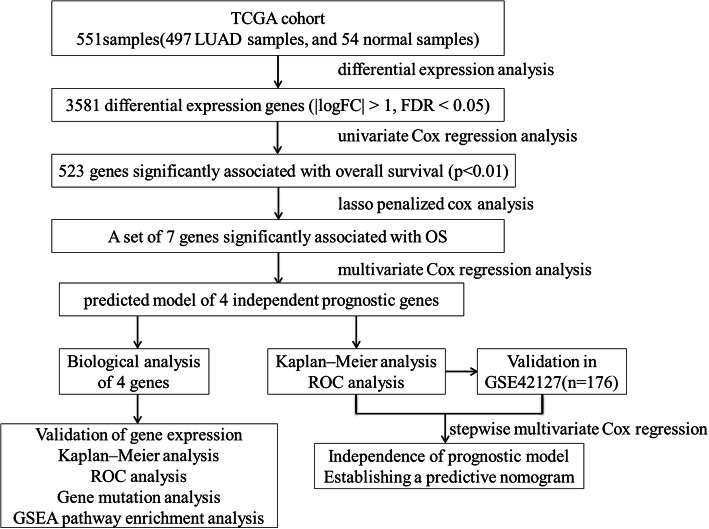
The flowchart showed the scheme of identifying and validating prognostic genes panel for lung adenocarcinoma in this study

A flowchart for our study was presented in Fig. 1. The lung adenocarcinoma mRNA sequencing dataset was downloaded from the TCGA database. A total of 3581 DEGs were obtained according to the criterion: |logFC| > 1, FDR < 0.05, including 2386 up-regulated genes and 1195 down-regulated genes. List, Heatmap, and volcano plot of the DEGs were shown in the supplementary document: Additional file 1, Additional file 2, Additional file 3.

### Establishment of a four-gene panel as a prognostic indicator

**Fig. 2:**
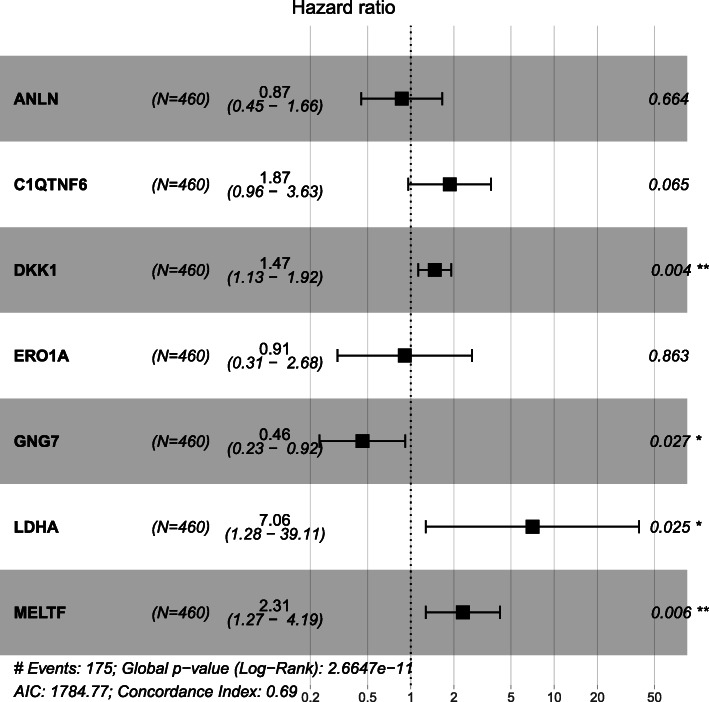
Forest plot of the multivariate Cox regression analysis establishing a four-gene panel as a prognostic indicator in LUAD

**Fig. 3:**
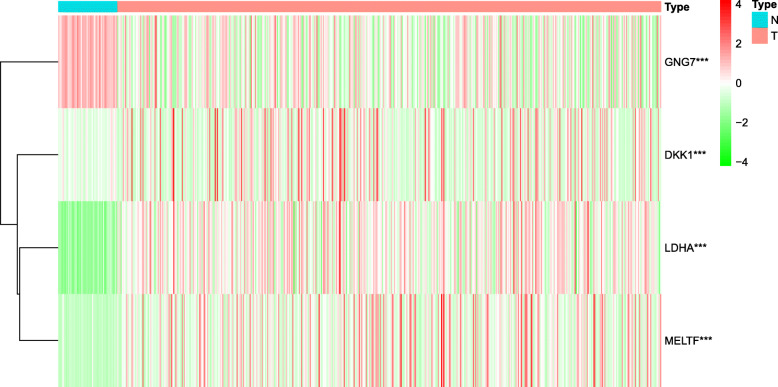
The heat map of differential expression of the four genes in the panel

**Fig. 4:**
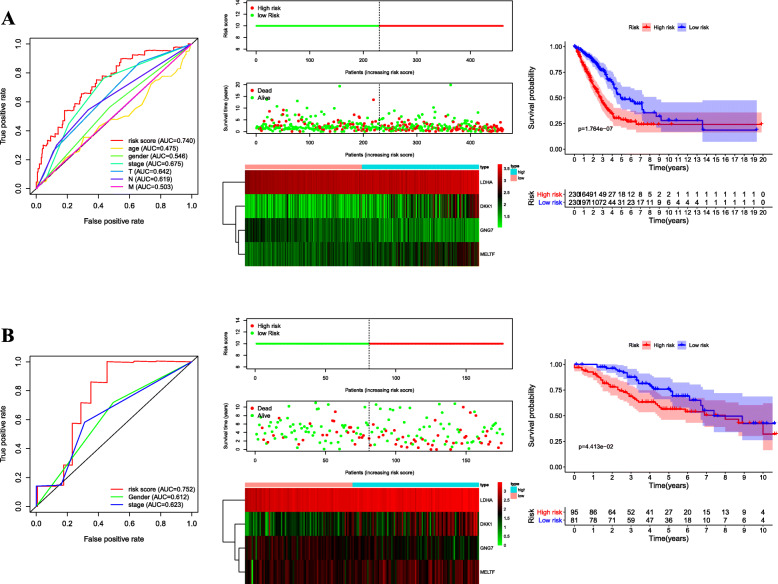
Time-dependent ROC analysis, risk score analysis, and Kaplan–Meier analysis for the four-gene panel in LUAD. **a** Time-dependent ROC analysis, risk score, heatmap of mRNA expression, and Kaplan–Meier curve of the four-gene panel in TCGA cohort. **b** Time-dependent ROC analysis, risk score, heatmap of mRNA expression, and Kaplan–Meier curve of the four-gene panel in GSE42127 cohort

Univariate Cox regression analysis was performed for identifying the DEGs associated with OS using the “survival” package of R language. Of the 3581 DEGs, 523 genes were identified as being associated with OS for LUAD patients (*p* < 0.01, Additional file 4). Then, lasso regression analysis was implemented to further obtain a stable set of genes (Additional file 5). Seven genes significantly associated with OS were screened out via this analysis (ANLN, C1QTNF6, DKK1, ERO1A, GNG7, LDHA, MELTF). At last, a four-gene panel as a prognostic indicator was obtained via multivariate Cox regression analysis. The forest map of Cox regression analysis was shown in Fig. 2. The four genes screened were dickkopf WNT signaling pathway inhibitor 1(DDK1), G protein subunit gamma 7(GNG7), lactate dehydrogenase A (LDHA), melanotransferrin (MELTF, also known as MTF1(metal regulatory transcription factor 1)). Among them, DKK1, LDHA and MELTF are high-expressed in tumor tissues compared with tissue adjacent to carcinoma, but GNG7 is low-expressed. The heat map of differential expression was shown in Fig. 3. The risk score = (0.38606 * Expression_DKK1_) + (− 0.77458 * Expression_GNG7_) + (1.95469 * Expression_LDHA_) + (0.83740 * Expression_MELTF_). Each patient from the TCGA-LUAD database was awarded a risk score based on the Cox regression model composed of the four genes. The results indicated that high-risk group had a worse prognosis compared with the low-risk group. The area under the ROC curve (AUC) of this four-gene panel as a prognostic indicator was 0.740 and was superior to other clinical indicators used for prognostic classification (Fig. 4a).

### Validation of the four-gene panel as a prognostic indicator

To further validate the accuracy of the four-gene panel as a prognostic indicator, we computed the risk score of each patient in GSE42127 using the same model. Consistent with previous results, a significantly worse OS was observed in the high-risk group compared with the low-risk group. ROC curve showed that the AUC for OS was 0.752, indicating a better predictive performance compared with other clinical indicators used for prognostic classification (Fig. 4b).

### Independent prognostic value of the four-gene panel

**Fig. 5:**
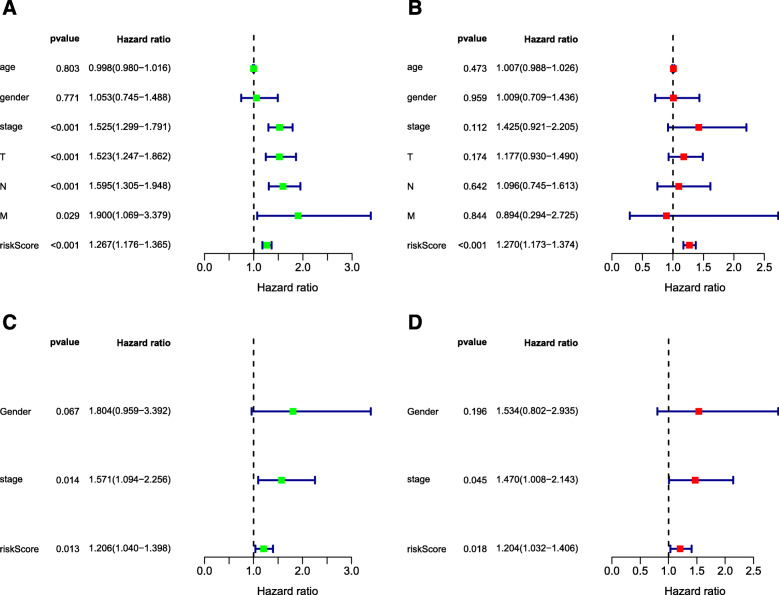
Forrest plot of the univariate and multivariate Cox regression analysis in LUAD. **a** Univariate Cox regression analysis for OS in TCGA-LUAD. **b** Multivariate Cox regression analysis for OS in TCGA-LUAD. **c** Univariate Cox regression analysis for OS in GSE42127. **d** Multivariate Cox regression analysis for OS in GSE42127

Three hundred forty-four patients from the TCGA-LUAD database with complete clinical information including age, gender, and TNM stage were included for further analysis. Univariate and Multivariate Cox regression analysis suggested that only the risk score calculated from the four-gene panel was independent prognostic factors for OS (HR = 1.270, *p* < 0.001). The consistent result was obtained in the patients from GSE42127 (HR = 1.204, *p* = 0.018). The results were presented in Fig. 5.

### Establishment of predictive nomogram

**Fig. 6:**
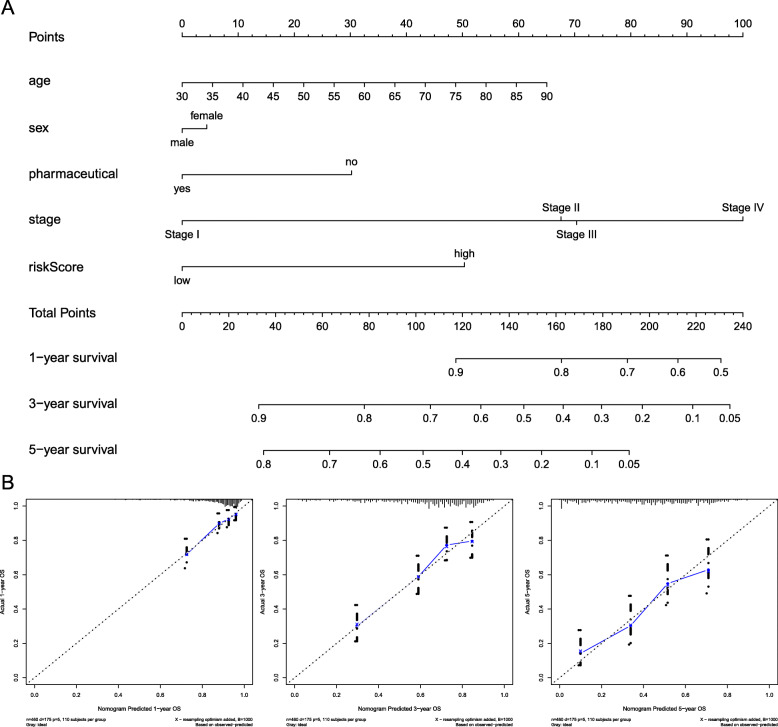
Nomogram predicting overall survival for LUAD patients. **a** For each patient, the points were calculated from the five predictors in the nomogram. The sum of these points is located on the‘Total Points’axis. Then a line is drawn downward to determine the possibility of 1-, 3-, and 5-year overall survival for LUAD. **b** The calibration curves for consistency validation of the nomogram. The X-axis represents nomogram-predicted OS and the Y-axis represents actual OS for 1, 3, 5 year. Dashed line at 45° represents perfect prediction and the actual performances of our nomogram are blue line. The more the blue lines and dashed lines in the graph coincide, the better the predictive performance of the nomogram

We established a nomogram to predict 1-year, 3-year, and 5-year OS in 460 patients with complete clinical information from the TCGA-LUAD database using five factors including risk score, age, sex, pharmaceutical, and pathologic stage (Fig. 6a). The C-index for the nomogram model was 0.710 (95% CI 0.624–0.796). Calibration curve showed that the nomogram had the superior prediction efficiency (Fig. 6b). These results indicated that the nomogram might be to serve as a prognostic model used for clinical management of LUAD patients.

### The genetic alteration, expression and survival analysis of the four genes

**Fig. 7:**
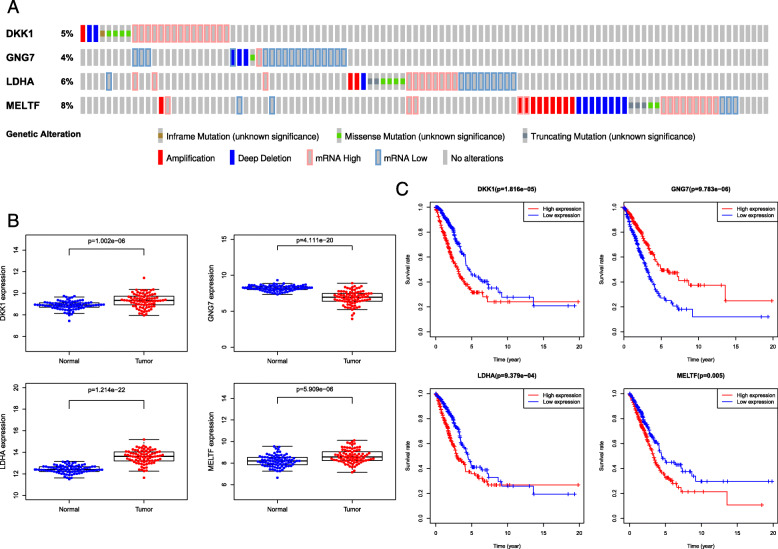
The genetic alteration, expression and survival analysis of the four genes. **a** The genetic alterations in the four genes. Each block represents a sample, and a different color represents a different form of genetic alteration. Data was obtained from the cBioportal (https://www.cbioportal.org/). **b** The four genes mRNA expression from GSE75037. **C** The survival analysis from TCGA-LUAD

We explored the genetic alteration of the four genes by using the mutation data obtained from the cBioPortal for Cancer Genomics (https://www.cbioportal.org/) [15, 16]. In this database, 9 % (21/230) of patients showed genetic alterations in the four genes. Missense mutation, amplification and deep deletion were common genetic alteration (Fig. 7a). We further validated the expression of the four genes using the lung adenocarcinoma dataset (GSE75037 [17]) from the GEO database, and the results were consistent with the analysis of the TCGA-LUAD dataset (Fig. 7b). Kaplan-Meier survival curves indicated that high expression of DKK1, LDHA, and MELTF and low expression of GNG7 were associated with a poor OS for LUAD (Fig. 7c).

### Predictive value of the four-gene panel for patients with EGFR, KRAS and TP53 mutation

**Fig. 8:**
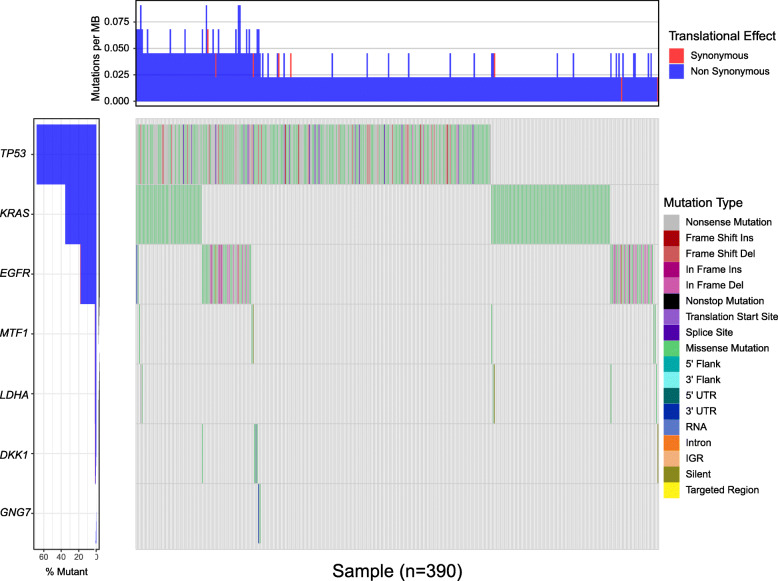
A heatmap of mutations in TP53, KRAS, EGFR and the four genes

**Fig. 9:**
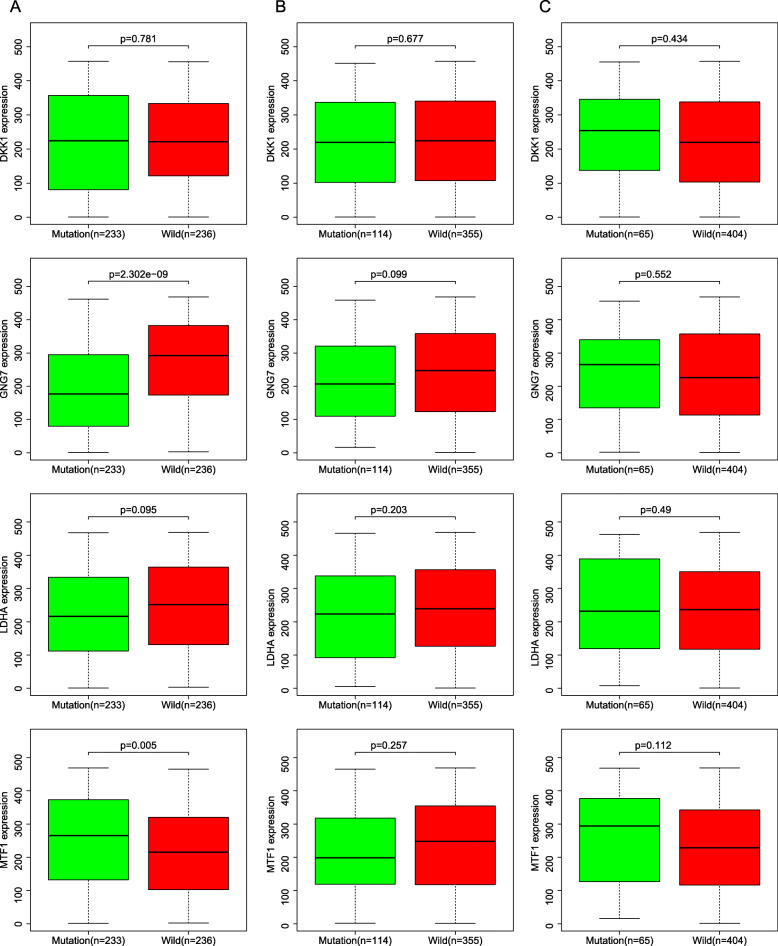
The expression of the four genes in different EGFR, KRAS and TP53 mutation

**Fig. 10:**
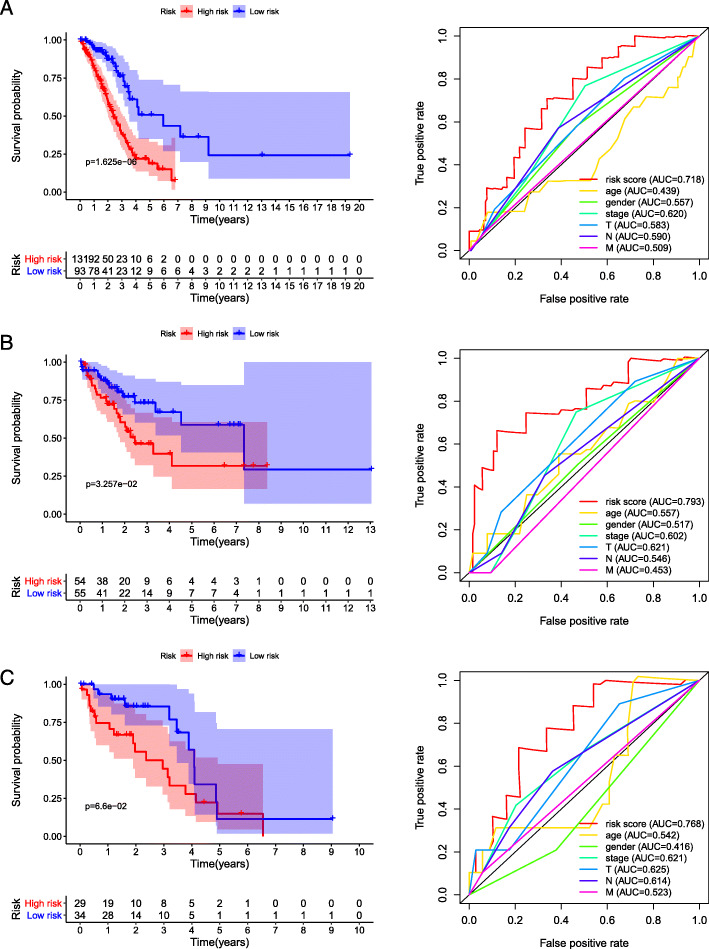
Predictive value of the four-gene panel for patients with EGFR, KRAS and TP53 mutation. **a** Kaplan–Meier curve and Time-dependent ROC curve for patients with TP53 mutation. **b** Kaplan–Meier curve and Time-dependent ROC curve for patients with KRAS mutation. **c** Kaplan–Meier curve and Time-dependent ROC curve for patients with EGFR mutation

To explore the predictive value of the four-gene panel for patients with EGFR, KRAS and TP53 mutation, we performed a combined analysis of gene mutation and transcription data. A heatmap of mutations in TP53, KRAS, EGFR and the four genes was shown in Fig. 8. The results showed that mRNA expression differences of GNG7 and MTF1 (MELTF) were only observed in TP53 mutant and wild-type patients (Fig. 9). We further analyzed the predictive value of the four-gene panel for OS in LUAD patients with EGFR, KRAS and TP53 mutations, respectively. The results showed that high-risk group had a worse OS in LUAD patients with TP53 mutations and KRAS mutations (*p* < 0.05). The AUC of this four-gene panel as a prognostic indicator was 0.718 and 0.793 in LUAD patients with TP53 and KRAS mutation, respectively. However, in patients with EGFR mutations, there was no significant difference in OS between the high-risk and low-risk groups (Fig. 10).

### Gene set enrichment analyses

**Fig. 11:**
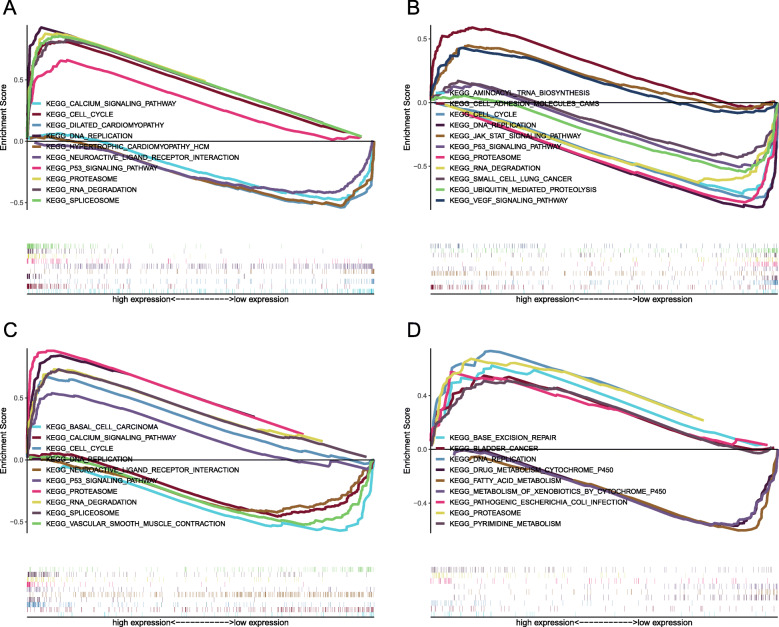
Enrichment plots from Gene Set Enrichment Analyses. **a** Gene Set Enrichment Analyses for DKK1. **b** Gene Set Enrichment Analyses for GNG7. **c** Gene Set Enrichment Analyses for LDHA. **d** Gene Set Enrichment Analyses for MELTF

GSEA analysis was used to identify signaling pathways enriched in low and high expression of the four genes, respectively. The results revealed that genes involved in cell cycle, ubiquitin mediated proteolysis, RNA degradation, aminoacyl tRNA biosynthesis, DNA replication, proteasome, small cell lung cancer, and P53 signaling pathway were enriched in GNG7 low expression group. In the high-expressed group of DKK1, KEGG pathways including cell cycle, RNA degradation, spliceosome, proteasome, DNA replication, P53 signaling pathway and so on were enriched. In the high-expressed group of LDHA, KEGG pathways including proteasome, RNA degradation, spliceosome, DNA replication, RNA polymerase, cell cycle, P53 signaling pathway and so on were enriched. In the high-expressed group of MELTF, the enriched KEGG pathways were mainly focused on bladder cancer, proteasome, DNA replication, base excision repair, pyrimidine metabolism. These results suggested that the absence of GNG7 expression and the increase of DKK1, LDHA and MELTF expression may be significantly related to the metabolism of genetic material, especially in the regulation of cell cycle pathway (Fig. 11).

### DNA methylation level and mRNA expression of GNG7

**Fig. 12:**
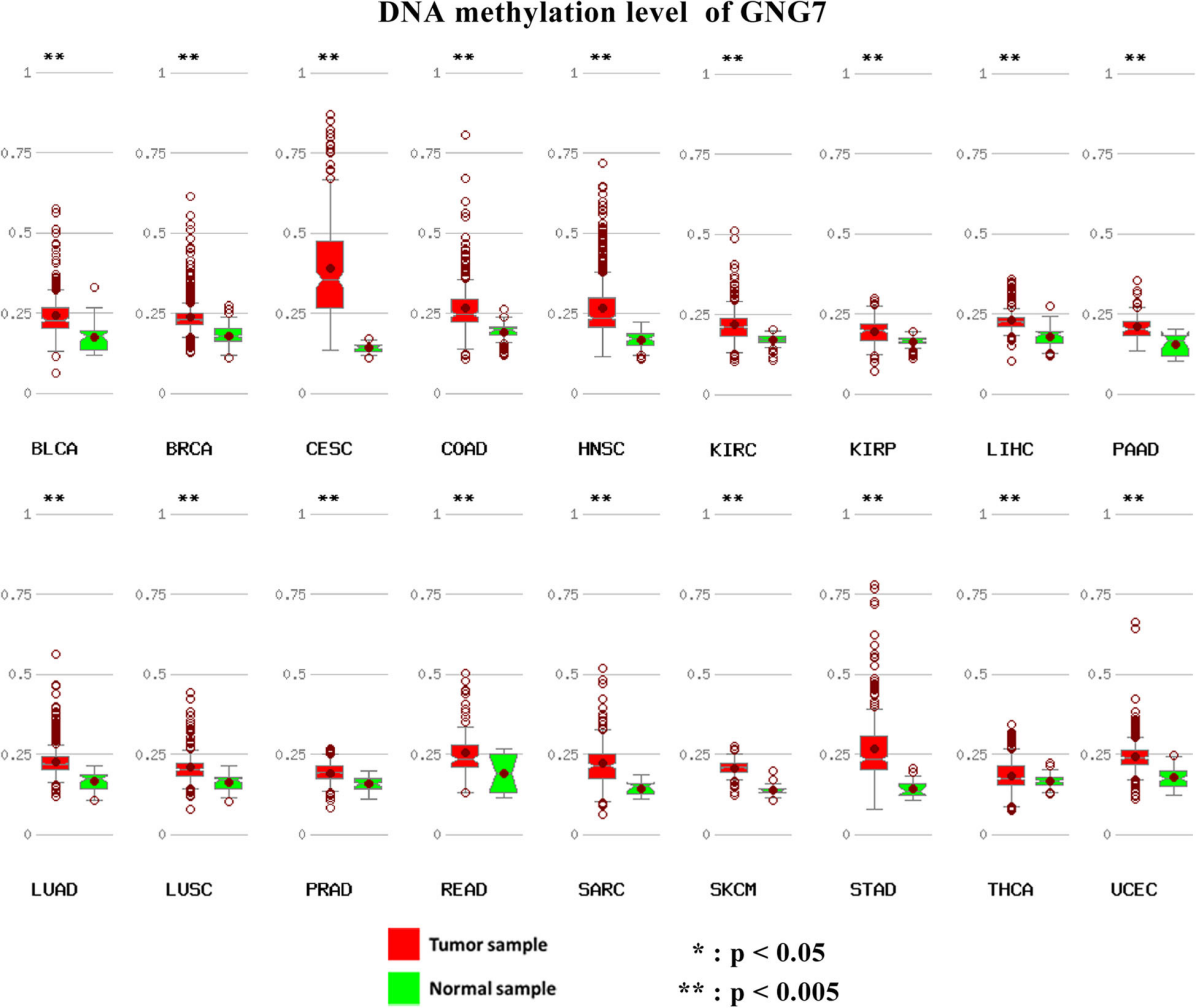
DNA methylation level of GNG7 in tumor and normal tissues. DNA methylation levels of GNG7 were significantly higher in 18 kinds of cancerous tissues than adjacent noncancerous tissues

**Fig. 13:**
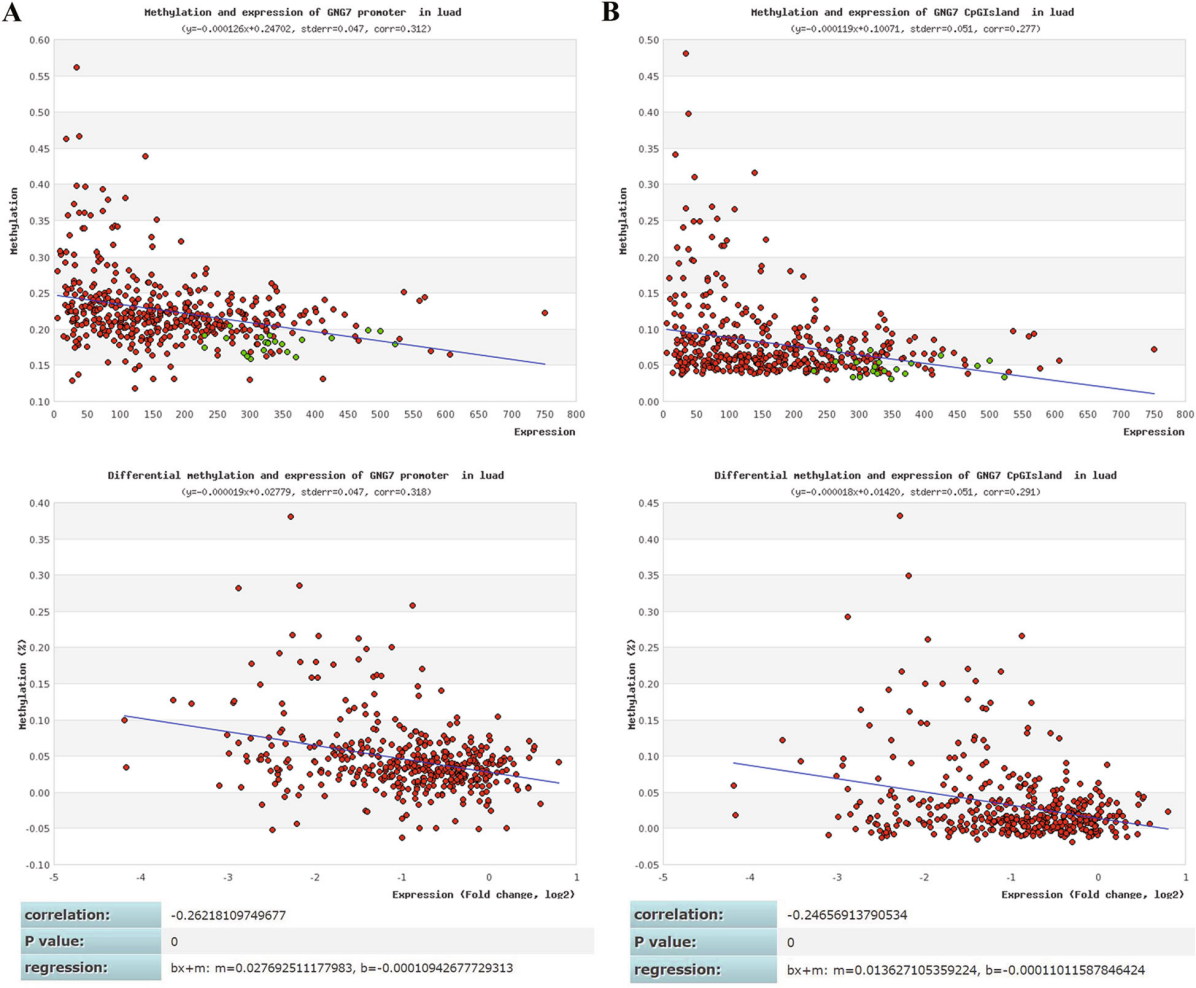
DNA methylation level and mRNA expression of GNG7. Methylation level of the promoter and CpG Island region was negatively correlated with mRNA expression of GNG7

We further explored the relationship between DNA methylation level and mRNA expression of GNG7 using MethHC database. The results showed that the DNA methylation levels of GNG7 were significantly higher in 18 kinds of cancerous tissues than adjacent noncancerous tissues (Fig. 12). Furthermore, methylation level of the promoter and CpG Island region was negatively correlated with mRNA expression of GNG7 (Fig. 13).

## Discussion

LUAD remains a serious threat to human health worldwide. Despite the fact that molecular targeted therapy and immunotherapy have made great progress, the OS of LUAD is still poor as the lack of accurate early diagnosis and prognosis markers. Owing to tumor heterogeneity, traditional clinical parameters such as TNM stage cannot meet the requirements of accuracy and individuation for prognostic prediction. Identification of accurate prognostic biomarkers and novel and effective therapeutic targets remains particularly urgent. And the combination of multiple prognostic genes seems to be more valuable and promising. Prognostic prediction models based on multiple genes combination have been established and validated in various cancers [7, 18, 19].

In the present study, we established a four-gene panel (including DKK1, GNG7, LDHA, and MELTF) as a prognostic prediction model for LUAD. Each patient from TCGA-LUAD obtained a risk score based on this model, and the risk score was an independent prognostic indicator of LUAD. Besides, the patients in high-risk score group shown poorer OS compared with patients in the low-risk score group. The consistent result was achieved in another independent cohort from the GEO database (GSE42127). The ROC curve demonstrated that the predictive performance of the risk score model as a prognostic indicator was superior both in the TCGA-LUAD cohort and in the GSE42127 cohort, compared with other clinical parameters. Nomogram combining risk score with other clinical parameters may be to serve as a prediction model used for clinical monitoring for OS in LUAD patients. All these results suggested that the prediction model based on the four-gene panel could be an effective and promising prognostic indicator for OS in LUAD patients.

DKK1, also named as DKK-1(dickkopf WNT signaling pathway inhibitor 1), has been proved to be differential expression in various tumors and participate in the regulation of growth, invasion, angiogenesis and metastasis of tumor [20–23]. In NSCLC, DKK1 be thought to be involved in tumor cell migration, invasion, and EMT processes, and could be used as an effective diagnostic and prognostic indicator and a potential therapeutic target [24–26]. LDHA (lactate dehydrogenase A), a crucial enzyme of energy metabolism, is elevated in various cancers compared with normal tissues. Previous studies showed that LDHA could promote tumor cells proliferation, invasion, migration, tumor progression, and metastasis, and might be a potential therapeutic target [27–31]. MELTF, also known as MTf (Melanotransferrin) or MTF1 (metal regulatory transcription factor 1), as an iron (Fe) binding transferrin homolog, is mainly expressed in melanoma and is low expression in normal tissues. Previous studies indicated that MTf plays a key role in cell invasion and migration [32, 33]. Subsequent studies indicated that it could promote carcinoma cell invasion, migration, proliferation, and EMT progression and be an attractive target [34–38].

GNG7 (G protein subunit gamma 7), a novel possible tumor suppressor gene, is proved to be down-regulated in various carcinoma, including head and neck squamous cell carcinoma, clear cell carcinoma of kidney, pancreatic cancer, oesophageal cancer, lung adenocarcinoma [39–43]. However, the mechanism of its role in tumorigenesis and progression is still little known. We further validated the expression of the four genes using the lung adenocarcinoma dataset GSE75037 dataset, and the results were consistent with the analysis of the TCGA-LUAD dataset. Kaplan-Meier survival curves indicated that high expression of DKK1, LDHA, and MELTF and low expression of GNG7 were associated with a poor OS for LUAD.

We further explored the predictive value of the four-gene panel for patients with EGFR, KRAS and TP53 mutation. The results showed that the expression of GNG7 is lower in TP53 mutant than wild-type patients, but expression of MELTF was the reverse. It is suggested that TP53 may play an opposite role in the expression regulation of the two genes. In addition, the four-gene panel was still excellent predictors for OS in LUAD patients with TP53 and KRAS mutations. It is suggested that the four-gene panel have useful predictive value and are not affected by mutations in these key genes.

The results of GSEA suggested that the absence of GNG7 expression and the increase of DKK1, LDHA and MELTF expression may be significantly related to the metabolism of genetic material, especially in the regulation of cell cycle pathway. This provides a sound theoretical basis for the future design of targeted therapy drugs for these 4 genes from the perspective of genetic material metabolism. DNA methylation is part of the common mechanisms of regulating genes expression. Our results showed that DNA methylation levels of the GNG7 were significantly higher in multiple tumors than in normal tissues. Furthermore, methylation level of the promoter and CpG Island region was negatively correlated with mRNA expression of GNG7. It indicated DNA methylation of GNG7 may involves in regulation of its expression.

Overall, our study established an accurate and effective four-gene panel prognostic model for OS in LUAD patients. Risk scores based on this four-gene panel can be used to determine the OS of LUAD patients. Nomogram combining our signature with clinical parameters like pharmaceutical, age, TNM stage can be utilized to predict 1-year, 3-year, and 5-year survival in LUAD patients. Therefore, it will be useful for prognosis and follow-up monitoring of LUAD patients and reducing the extra cost for molecular diagnosis such as whole-genome sequencing. Besides, as a possible novel tumor suppressor gene, the elucidating mechanism of GNG7 in tumor genesis and progression will deepen our understanding of carcinomas including lung cancer and have great theoretical and scientific significance. However, it should be noted that there are still some limitations to our study. Firstly, the data in our study mainly came from TCGA and GEO databases, and it was necessary to further verify the expression and prognostic value of the four genes at mRNA and protein level in an large independent clinical cohort. Secondly, the nomogram requires further external calibration and validation to improve predictive effectivity and accuracy. Thirdly, the potential biological mechanisms of the four genes in LUAD need to be further illuminated using functional studies.

## Conclusions

Our study proposed a novel four-gene panel and nomogram to predict the OS for patients with LUAD, which may contribute to predicting prognosis accurately and making clinical decisions of individual therapy for LUAD patients. The four genes may be significantly related to the metabolism of genetic material, especially in the regulation of cell cycle pathway. This provides a reliable theoretical basis for the future design of targeted therapy drugs for these 4 genes from the perspective of genetic material metabolism.

## Supplementary Information

**Additional file 1.** List of differentially expressed genes.

**Additional file 2: Figure S1.** Heatmap of differentially expressed genes.

**Additional file 3: Figure S2.** Volcano plot of differentially expressed genes.

**Additional file 4.** List of differentially expressed genes associated with OS for LUAD.

**Additional file 5: Figure S3.** LASSO profiles of the 523 prognostic genes in LUAD. (A) LASSO coefficient profiles of the 523 prognostic genes in LUAD. (B) Lasso deviance profiles of the 523 prognostic genes in LUAD.

## Data Availability

The datasets supporting the conclusions of this article are available in the TCGA-GDC (https://portal.gdc.cancer.gov/) repository.

## Abbreviations

OS: Overall survival
LUAD: Lung adenocarcinoma
TCGA: The Cancer Genome Atlas
LASSO: Least Absolute Shrinkage and Selection Operator
ROC: Receiver operating characteristic
GEO: Gene Expression Omnibus
GSEA: Gene set enrichment analysis
TCGA-LIHC: The Cancer Genome Atlas-Liver Hepatocellular Carcinoma Dataset
TCGA-LUAD: The Cancer Genome Atlas-Lung Adenocarcinoma Dataset
DEGs: Differential expression genes
C-index: The concordance index
MethHC: A database of DNA Methylation and gene expression in Human Cancer
FDR: False discovery rate
DDK1: Dickkopf WNT signaling pathway inhibitor 1
GNG7: G protein subunit gamma 7
LDHA: Lactate dehydrogenase A
MELTF: Melanotransferrin
MTF1: Metal regulatory transcription factor 1
AUC: Area under the ROC curve
