# Supplementary figures and images for: Identification of a four-gene panel predicting overall survival for lung adenocarcinoma
Source: BMC Cancer. 2020 Dec 7;20:1198. doi: 10.1186/s12885-020-07657-9 (PMC7720456; doi:10.1186/s12885-020-07657-9)

# Volcano

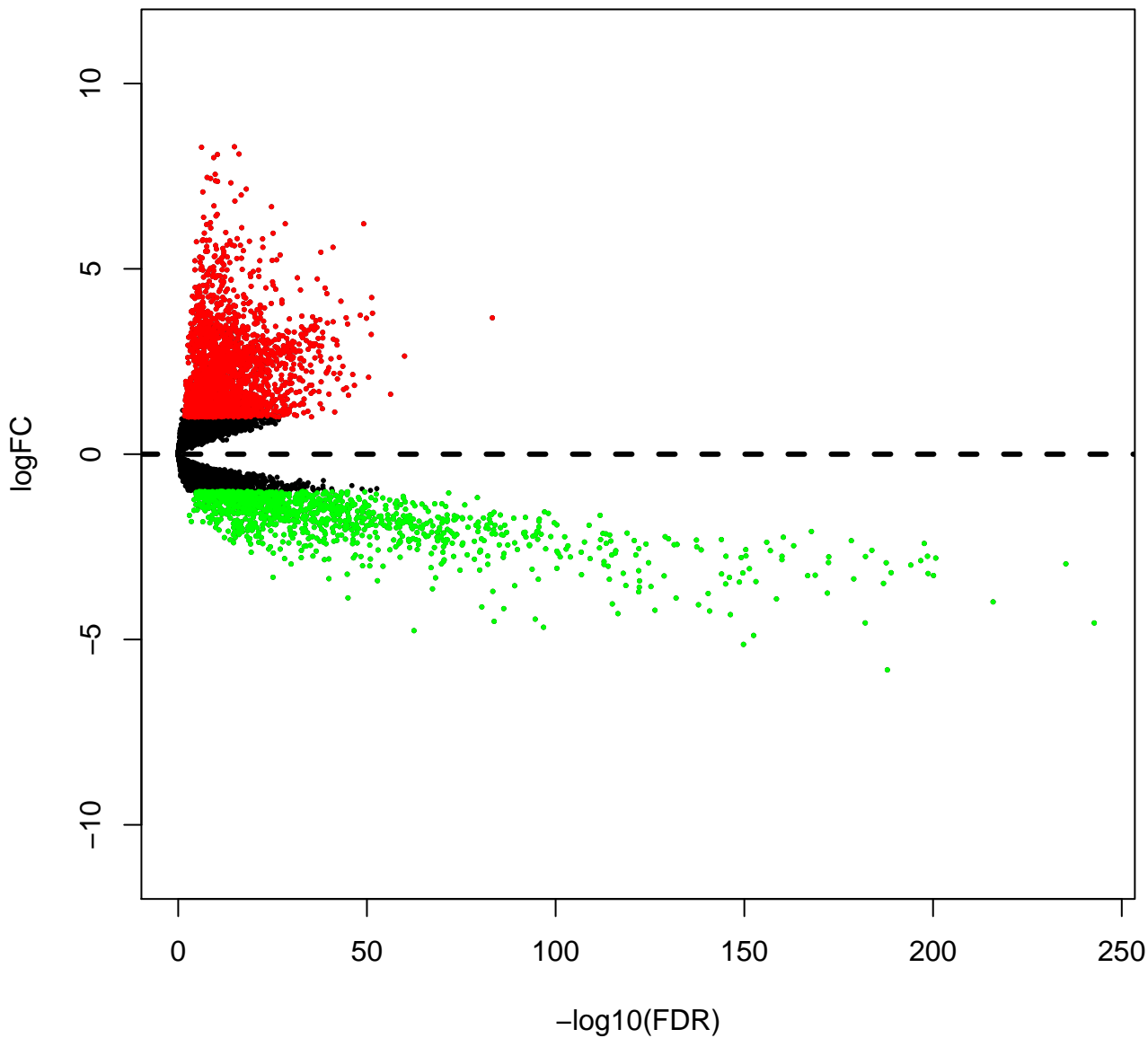

Supplement: Supplementary file 3 — Additional file 3: Figure S2. Volcano plot of differentially expressed genes. [file 12885_2020_7657_MOESM3_ESM.pdf]

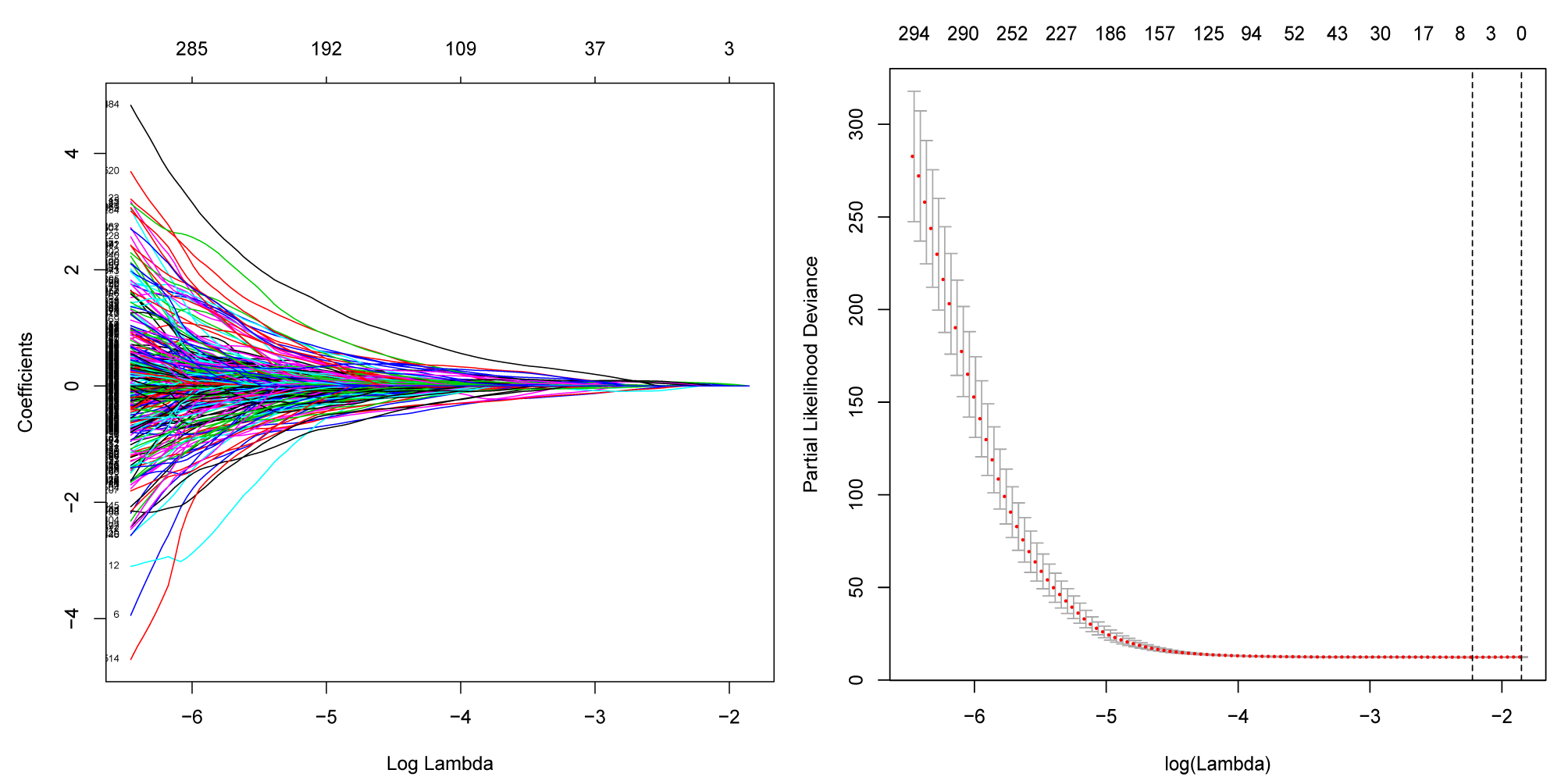

Supplement: Supplementary file 5 — Additional file 5: Figure S3. LASSO profiles of the 523 prognostic genes in LUAD. (A) LASSO coefficient profiles of the 523 prognostic genes in LUAD. (B) Lasso deviance profiles of the 523 prognostic genes in LUAD. [file 12885_2020_7657_MOESM5_ESM.tif]
