# Supplementary material for: Identification of a four-gene panel predicting overall survival for lung adenocarcinoma
Source: BMC Cancer. 2020 Dec 7;20:1198. doi: 10.1186/s12885-020-07657-9 (PMC7720456; doi:10.1186/s12885-020-07657-9)
Supplement: Supplementary file 2 — Additional file 2: Figure S1. Heatmap of differentially expressed genes. [file 12885_2020_7657_MOESM2_ESM.pdf]

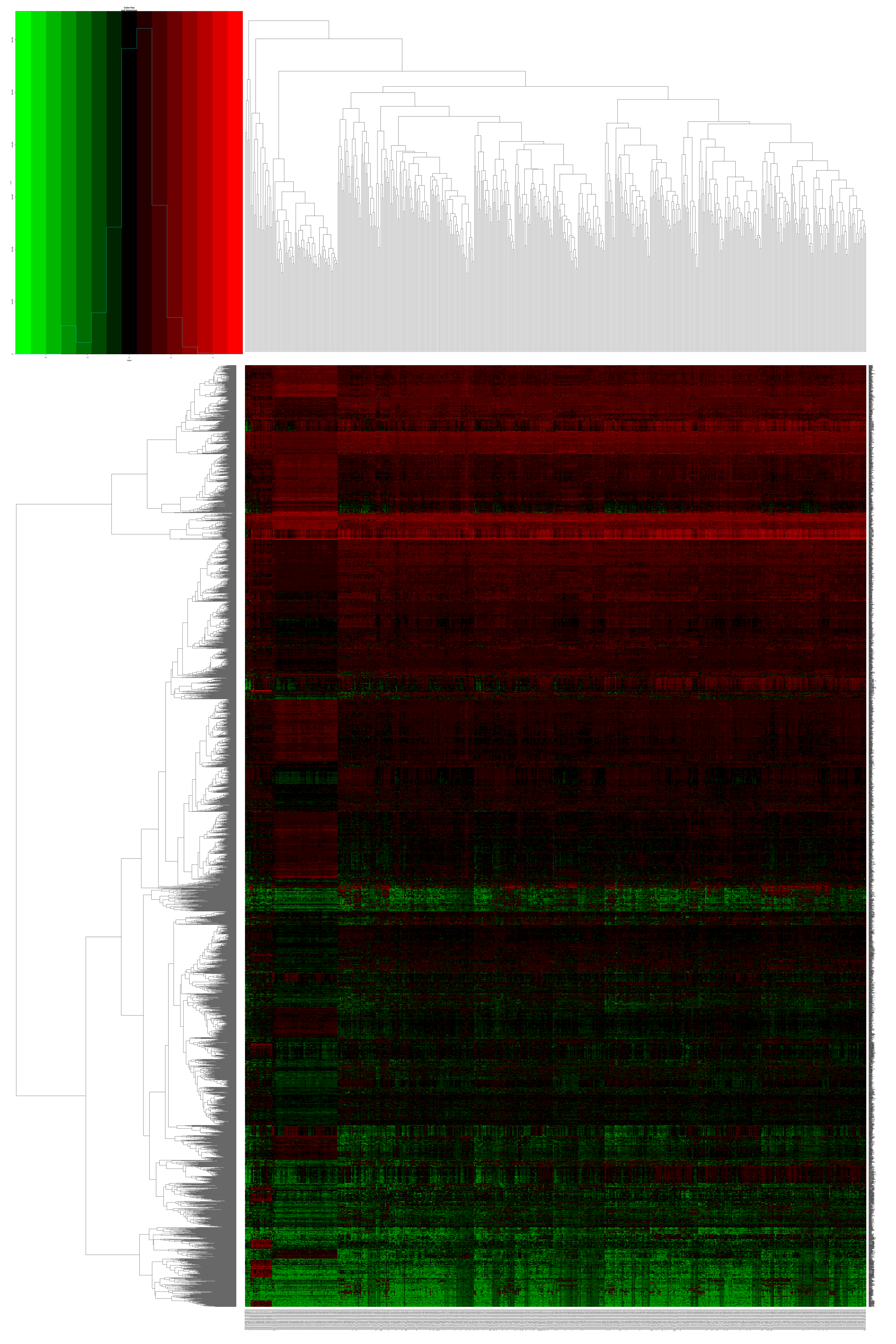
Color Key
and Histogram
Count
0
1e+05
2e+05
3e+05
4e+05
5e+05
6e+05
−4
−2
0
2
4
Value
